# The societal economic burden of autosomal dominant polycystic kidney disease in the United States

**DOI:** 10.1186/s12913-020-4974-4

**Published:** 2020-02-18

**Authors:** Martin Cloutier, Ameur M. Manceur, Annie Guerin, Myrlene Sanon Aigbogun, Dorothee Oberdhan, Marjolaine Gauthier-Loiselle

**Affiliations:** 1 Analysis Group, Inc., Montréal, QC Canada; 2 Otsuka Pharmaceutical Development & Commercialization, Inc., Princeton, NJ USA

**Keywords:** Autosomal dominant polycystic kidney disease, Burden of disease, Societal impact, Direct costs, Indirect costs

## Abstract

**Background:**

Autosomal dominant polycystic kidney disease (ADPKD) is one of the most common inherited kidney diseases characterized by progressive development of renal cysts and numerous extra-renal manifestations, eventually leading to kidney failure. Given its chronic and progressive nature, ADPKD is expected to carry a substantial economic burden over the course of the disease. However, there is a paucity of evidence on the impact of ADPKD from a societal perspective. This study aimed to estimate the direct and indirect costs associated with ADPKD in the United States (US).

**Methods:**

A prevalence-based approach using data from scientific literature, and governmental and non-governmental organizations was employed to estimate direct healthcare costs (i.e., medical services, prescription drugs), direct non-healthcare costs (i.e., research and advocacy, donors/recipients matching for kidney transplants, transportation to/from dialysis centers), and indirect costs (i.e., patient productivity loss from unemployment, reduced work productivity, and premature mortality, caregivers’ productivity loss and healthcare costs). The incremental costs associated with ADPKD were calculated as the difference between costs incurred over a one-year period by individuals with ADPKD and the US population. Sensitivity analyses using different sources and assumptions were performed to assess robustness of estimates and account for variability in published estimates.

**Results:**

The estimated total annual costs attributed to ADPKD in 2018 ranged from $7.3 to $9.6 billion in sensitivity analyses, equivalent to $51,970 to $68,091 per individual with ADPKD. In the base scenario, direct healthcare costs accounted for $5.7 billion (78.6%) of the total $7.3 billion costs, mostly driven by patients requiring renal replacement therapy ($3.2 billion; 43.3%). Indirect costs accounted for $1.4 billion (19.7%), mostly driven by productivity loss due to unemployment ($784 million; 10.7%) and reduced productivity at work ($390 million; 5.3%). Total excess direct non-healthcare costs were estimated at $125 million (1.7%).

**Conclusions:**

ADPKD carries a considerable economic burden, predominantly attributed to direct healthcare costs, the majority of which are incurred by public and private healthcare payers. Effective and timely interventions to slow down the progression of ADPKD could substantially reduce the economic burden of ADPKD.

## Background

Autosomal dominant polycystic kidney disease (ADPKD) is the most common of the inherited renal cystic diseases, a group of related but pathologically distinct disorders characterized by the progressive development of renal cysts [1]. The course of the disease is often associated with various other systemic extra-renal manifestations, including cysts in other organs such as the liver, seminal vesicles, pancreas, arachnoid membrane, and other complications such as urinary tract infection, acute or chronic flank and abdominal pain, and cardiovascular abnormalities, including hypertension, peripheral vascular disease, mitral valve prolapse, and intracranial aneurysms, which contribute to a greater mortality risk [1, 2]. The progressive irreversible decline in kidney function eventually leads to kidney failure [3]. In the United States (US), ADPKD is the fourth leading cause of end-stage renal disease (ESRD) [1, 4], with ESRD occurring at an average age of 57 years among individuals with ADPKD, leading to a substantial population of patients requiring expensive dialysis or renal replacement therapy [5].

Given that ADPKD is a life-long and progressive disorder, a substantial economic burden over the course of the disease is expected, including a variety of treatment-related and other costs [6–13]. Few studies to date have estimated direct and indirect costs among patients with ADPKD. Studies in the US have predominantly focused on direct medical costs, and found that healthcare costs tended to increase with advancing disease stage and were particularly high among patients with advanced renal dysfunction, including individuals with ESRD requiring renal replacement therapy (ESRD-RRT) [8, 10, 11]. To our knowledge, the only study that estimated both direct and indirect costs associated with ADPKD was based on a Nordic population (i.e., Denmark, Finland, Norway, and Sweden) [9], and may therefore not be representative of the economic burden of ADPKD in the US.

The economic burden of ADPKD from a US societal perspective remains unknown. With rising healthcare costs, there is a growing need for comprehensive estimates of the economic burden of ADPKD to inform clinical practice and decision-making.

Therefore, the objective of this study was to estimate the economic burden of ADPKD from a US societal perspective using the published literature and government and non-profit data sources.

## Methods

### Study design

The economic burden associated with ADPKD was estimated for 2018 and comprised direct healthcare costs, direct non-healthcare costs, and indirect costs. Cost components were identified from scientific publications, governmental agencies, and non-governmental organizations. Costs were stratified by disease stage when information was available.

Costs associated with ADPKD were calculated using a prevalence-based approach with a prevalence of 0.043% and the estimated 2018 US adult population from the US Census Bureau [14, 15]. The number of individuals with ESRD-RRT due to a primary diagnosis of ADPKD in the US was derived from the United States Renal Data System (USRDS) [16]. The distribution of the prevalence of ADPKD for chronic kidney disease (CKD) stages 1–5 was estimated based on the relative proportion of individuals with each disease stage (excluding individuals with ESRD-RRT) based on the literature [17]. The incremental costs associated with ADPKD were estimated based on the average cost difference between an individual with ADPKD and an individual from the US population (comparison group), and thus constitute the “excess” costs attributed to ADPKD; similar methodology has been used in several previous publications [18–26]. All costs were expressed in 2018 US dollars, with direct healthcare costs adjusted using the Consumer Price Index for All Urban Consumers (CPI-U): Medical Care and direct non-healthcare and indirect costs adjusted using the CPI-U: All Items [27]. Adjustments for population growth were made for parameters estimated prior to 2018 using the population estimates from the US Census Bureau [15].

### Direct healthcare costs

Excess direct healthcare costs associated with ADPKD were calculated based on the average annual excess direct healthcare costs incurred by an individual with ADPKD compared to those incurred by an individual from the US population, stratified by disease stage, multiplied by the estimated number of individuals with ADPKD by disease stage in the US [16, 17]. Excess direct healthcare costs incurred by payers for an insured individual were based on estimates from Knight et al. [10]. As reported by Knight et al., the comparison group of individuals from the US population consisted of a random sample of sex- and age-matched patients without polycystic kidney disease (PKD) or ADPKD [10]. To estimate the direct healthcare costs from a societal perspective, out-of-pocket expenditures were added as a proportion of the absolute costs based on estimates available in the literature for patients with non-dialysis dependent CKD, patients with ESRD, and the US population [16, 28–30]. In the absence of published estimates for the excess costs incurred by publicly insured and uninsured individuals with ADPKD, excess costs were assumed to be the same for privately and publicly insured individuals with ADPKD, and uninsured individuals with ADPKD were assumed to incur no excess costs compared to uninsured individuals in the US population. Excess direct healthcare costs by insurance type were weighted by the proportion of individuals by insurance type [16, 29] to obtain the estimated average excess direct healthcare costs for an individual with ADPKD compared to an individual from the US population, stratified by disease stage.

### Direct non-healthcare costs

Direct non-healthcare cost components included research, training, advocacy, matching of donors and recipients for kidney transplant, and transportation to and from dialysis centers. Costs of research, training, and advocacy were drawn directly from the estimates of funding for research on PKD reported by the National Institute of Health (NIH) and from the annual report of the PKD Foundation [31, 32]. Costs of matching donors and recipients for kidney transplant among individuals with ADPKD were based on the Organ Procurement and Transplantation Network (OPTN) fees and the United Network for Organ Sharing (UNOS) fees multiplied by the number of individuals waiting for a kidney transplant due to ADPKD [16, 33–35]. Costs of transportation to and from dialysis centers among individuals with ADPKD in the US were based on the average transportation costs for individuals using in-center dialysis multiplied by the number of individuals with ADPKD using in-center dialysis in the US [12, 16]. The number of individuals with ADPKD using in-center dialysis in the US was computed as the number of individuals with ESRD-RRT requiring dialysis due to cystic kidney disease in the US multiplied by the proportion of individuals with ESRD-RRT due to ADPKD among individuals with cystic kidney disease and the proportion of individuals requiring in-center dialysis among individuals requiring dialysis due to cystic kidney disease [16]. Of note, it was assumed that direct non-healthcare costs incurred by patients with ADPKD would be zero if these individuals did not have ADPKD. Therefore, the direct non-healthcare costs associated with ADPKD were assumed to be equal to the excess direct non-healthcare costs.

### Indirect costs

The studied components of indirect costs were reduced productivity at work, loss of productivity due to unemployment, loss of productivity from premature mortality, and caregiver burden, as previously identified by Cloutier et al. [18]. The latter included loss of productivity as a result of caregiving as well as incremental healthcare costs incurred by caregivers.

#### Unemployment

Costs associated with productivity loss from unemployment were estimated based on the excess number of unemployed individuals with ADPKD multiplied by the age-adjusted average annual wage in the US employed population [9, 16, 36]. The excess number of unemployed individuals with ADPKD was computed based on the number of individuals with ADPKD aged 15–64 years old by disease stage, the employment-to-population ratio in the ADPKD population by disease stage, and employment-to-population ratio in the US population given the average age of working-age individuals with ADPKD in each disease stage.

#### Reduced productivity at work

Reduced productivity at work was assessed based on the productivity weight in the ADPKD employed population by disease stage compared to the US population. Costs associated with this reduced productivity were further calculated based on the number of employed individuals with ADPKD by disease stage and the average annual wage in the US employed population given the average age of working-age individuals with ADPKD in each disease stage. The number of employed individuals with ADPKD was based on the number of individuals with ADPKD by disease stage and the employment-to-population ratio in the ADPKD population by disease stage [9, 36–38].

#### Premature mortality

Premature all-cause mortality is associated with a productivity loss for the society. These costs were estimated using the following components by age group: (1) all-cause excess mortality rate in the ADPKD population compared to the US general population [15, 39], (2) the average number of years of productive life (i.e., based on retirement age) in the US general population [40], and (3) the average annual wage in the US employed population [41]. In order to calculate the net present value of costs arising in the future, a 3% discount rate was applied to the productivity loss from premature all-cause mortality [42].

#### Caregiver burden

The productivity loss from caregiving was defined as the number of unpaid hours of care received by individuals with ADPKD [9]. The associated costs were calculated for each disease state by multiplying the number of hours lost by the average hourly wage in the US [36]. Of note, it was assumed that productivity loss due to caregiving would be zero if individuals with APDKD did not have ADPKD. Accordingly, for this component, the total costs were assumed to be equal to the excess costs.

Moreover, to obtain excess healthcare costs incurred by caregivers of individuals with ADPKD, the estimated excess direct healthcare costs incurred by a family caregiver compared to those of a non-caregiver were multiplied by the number of individuals with ADPKD receiving assistance from family caregivers by disease stage [29, 34, 43, 44]. The proportion of excess direct healthcare costs incurred by caregivers was derived from Albert et al. based on stratified analyses comparing caregivers and non-caregivers within groups defined by age, gender, and white-collar versus blue-collar status [43]. The excess direct healthcare costs incurred by a family caregiver were then computed by multiplying the proportion of excess costs among caregivers with the average direct all-cause healthcare costs per individual per year in the US population reported in the Medical Expenditure Panel Survey (MEPS) survey [29].

### Sensitivity analyses

Four sensitivity analyses were conducted to assess the impact of using different sources and assumptions on the estimates. First, an adjustment was made to the estimated excess direct healthcare costs among publicly insured individuals to reflect potentially higher excess direct healthcare costs compared to those of commercially insured individuals [45]. Second, direct healthcare costs for uninsured individuals with ADPKD were estimated based on the average medical costs reported in the literature for the US uninsured population and the ratio of the direct healthcare costs for individuals with ADPKD versus without ADPKD, as measured in Knight et al. [10, 18, 46]. Third, the number of hours per year devoted to caregiving for an individual with ADPKD was varied based on estimates for the CKD population [13, 47]. Finally, the economic burden of ADPKD was calculated based on the distribution of the prevalence of ADPKD per disease stage from Neumann et al., without adjustment for the number of individuals with ESRD-RRT based on the USRDS [17].

## Results

**Fig. 1:**
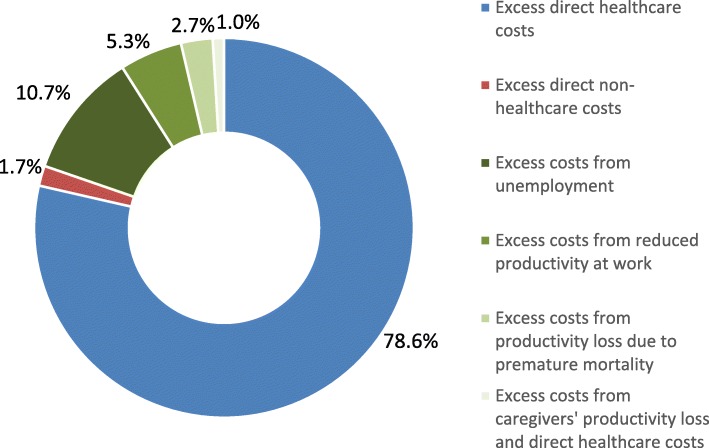
Distribution of total excess costs associated with ADPKD in the US in 2018. ADPKD: autosomal dominant polycystic kidney disease; US: United States

The total ADPKD population in the US in 2018 was estimated at 140,598 individuals based on a prevalence of 0.043%. The economic burden associated with ADPKD for these individuals was estimated at $7.3 billion in 2018, that is $51,970 per individual with ADPKD (Table 1). The main cost drivers were excess direct healthcare costs ($5.7 billion; 78.6%), followed by unemployment ($784 million; 10.7%) and reduced productivity at work ($390 million; 5.3%) (Fig. 1).

**Table 1:** Societal costs associated with ADPKD in the US in 2018

Component^1^	Excess costs (2018 USD)
Excess direct healthcare costs	
CKD stage 1	$545,894,174
CKD stage 2	$612,261,195
CKD stage 3	$619,300,610
CKD stage 4	$354,143,587
CKD stage 5 (excluding ESRD-RRT)	$442,190,064
ESRD-RRT	$3,167,501,412
Total excess direct healthcare costs	**$5,741,291,041**
Excess direct non-healthcare costs	
Research, training, and advocacy	$35,714,993
Costs of matching donors and recipients for kidney transplant	$7,583,334
Costs of transportation to and from dialysis center	$81,428,941
Total excess direct non-healthcare costs	**$124,727,268**
Excess indirect costs	
Unemployment	**$783,819,493**
CKD stages 1–3	$256,201,951
CKD stages 4–5 (excluding ESRD-RRT)	$115,955,070
ESRD-RRT	$411,662,472
Reduced productivity at work	**$390,316,213**
CKD stages 1–3	$237,533,624
CKD stages 4–5 (excluding ESRD-RRT)	$59,850,579
ESRD-RRT	$92,932,010
Productivity loss from premature mortality	**$196,789,459**
Caregivers’ productivity loss	**$53,060,981**
CKD stages 1–3	$7,086,663
CKD stages 4–5 (excluding ESRD-RRT)	$9,689,005
ESRD-RRT	$36,285,312
Caregivers’ direct healthcare costs	**$16,831,670**
CKD stages 1–3	$7,712,428
CKD stages 4–5 (excluding ESRD-RRT)	$1,210,672
ESRD-RRT	$7,908,570
Total excess indirect costs	**$1,440,817,816**
Total excess direct healthcare, direct non-healthcare, and indirect costs	**$7,306,836,126**

*ADPKD* autosomal dominant polycystic kidney disease; *CKD* chronic kidney disease; *CPI* consumer price index; *ESRD-RRT* end stage renal disease requiring renal replacement therapy; *US* United States; *USD* United States dollars

Notes

1. Based on a prevalence of 0.043%, the ADPKD population in the US was estimated at 140,598 individuals in the US. Of those, there were 29,669 individuals with CKD stage 1; 35,129 with CKD stage 2; 29,123 with CKD stage 3; 10,739 with CKD stage 4; 4004 with CKD stage 5 (excluding ESRD-RRT); and 31,934 with ESRD-RRT. Costs were adjusted to 2018 dollars using the Consumer Price Index

### Direct healthcare costs

**Fig. 2:**
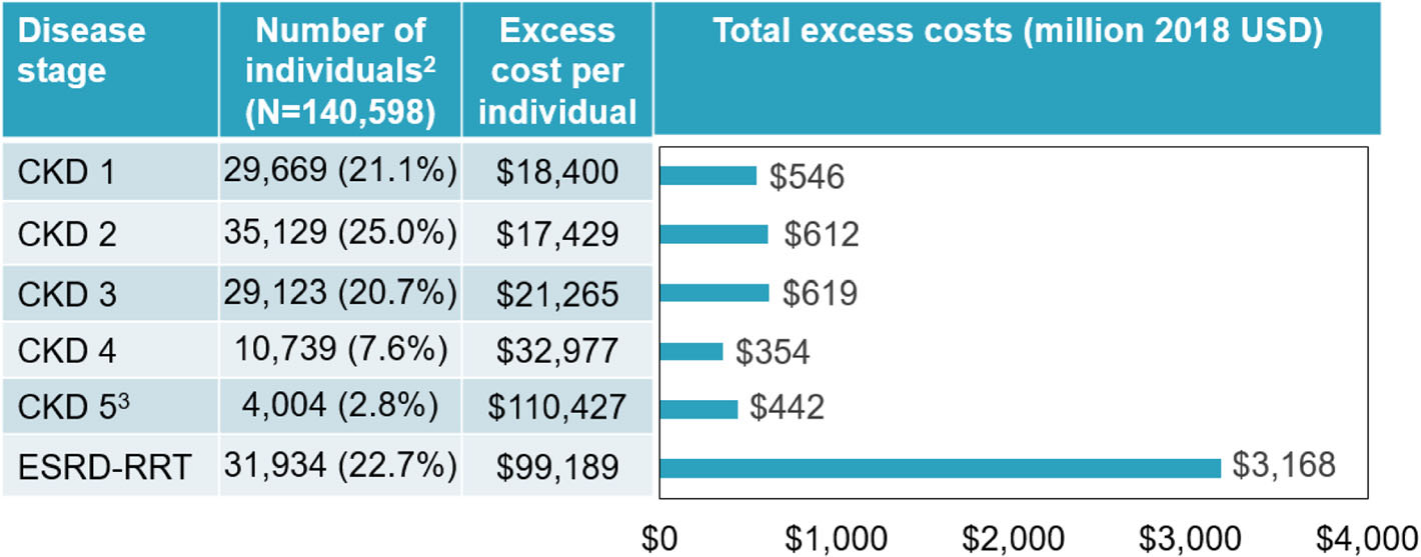
Direct healthcare costs^1^. ADPKD: autosomal dominant polycystic kidney disease; CKD: chronic kidney disease; ESRD-RRT: end stage renal disease requiring renal replacement therapy; US: United States. Notes: 1. As reported by Knight et al., patients with ADPKD were matched to a random sample of patients without polycystic kidney disease or ADPKD based on sex and age [10]. 2. Individuals with ADPKD in the US in 2018. 3. Excluding ESRD-RRT

The estimated annual excess direct healthcare costs for an individual with ADPKD compared to an individual from the US population increased with disease stage, with the difference ranging from < $20,000 for CKD stages 1 and 2 to > $110,000 for CKD 5 (Fig. 2). Out-of-pocket expenditures were assumed to be 11.2% of total direct healthcare costs for CKD stages 1–5, 5.7% for ESRD-RRT, and 12.2% for the US population [16, 28–30]. For CKD stages 1–5, the proportion of uninsured individuals was estimated at 8.1% [29], and that of Medicare-insured individuals was assumed to be that of individuals above 65 years old (i.e., 4.5 to 39.5%, across CKD stages). For ESRD-RRT, the proportion of uninsured and Medicare-insured individuals was estimated at 5.1 and 42.0%, respectively [16]. Overall, excess direct healthcare costs were estimated at $5.7 billion, 55.2% of which was incurred by individuals with ESRD-RRT.

### Direct non-healthcare costs

Costs of research, training, and advocacy for ADPKD in the US were estimated at $35.7 million, that is, $29.2 million for NIH-funded research and $6.6 million for the PKD Foundation. Costs of matching donors and recipients for kidney transplant among individuals with ADPKD were estimated at $7.6 million, that is, $979 per individual with ADPKD waiting for a kidney transplant (*n* = 7746). Costs of transportation to and from dialysis centers among individuals with ADPKD in the US were estimated at $81.4 million, that is $9112 per individual with ADPKD using in-center dialysis (*n* = 8936). Collectively, excess direct non-healthcare costs were estimated at $124.7 million.

### Indirect costs

Overall, annual excess indirect costs associated with ADPKD were estimated at $1.4 billion, 35.0% of which was associated with excess unemployment and reduced productivity at work among individuals with ESRD-RRT.

**Fig. 3:**
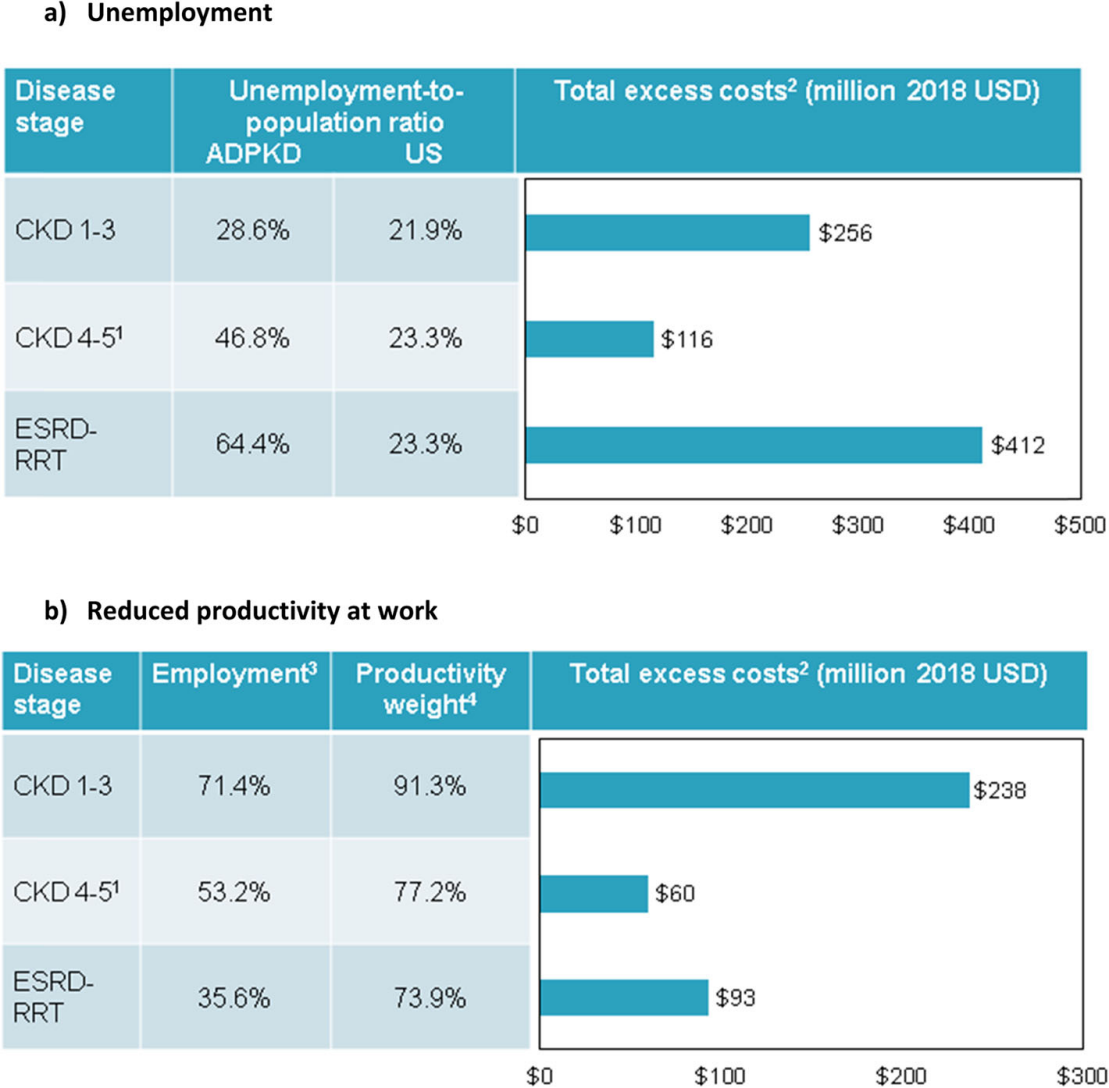
Indirect costs from unemployment and reduced productivity at work. ADPKD: autosomal dominant polycystic kidney disease; CKD: chronic kidney disease; ESRD-RRT: end stage renal disease requiring renal replacement therapy; US: United States. Notes: 1. Excluding ESRD-RRT. 2. Unemployment-to-population ratio and average annual earnings in the US population were age-adjusted given the average age of working-age individuals in each of the ADPKD disease stages. 3. Employment-to-population ratio among individuals with ADPKD. 4. Productivity weight in employed individuals with ADPKD (based on Work Productivity and Activity Impairment [WPAI])

The estimated difference in the employment-to-population ratio in the ADPKD and US populations increased with disease stage, and ranged from 6.7% for CKD stages 1–3 to 41.1% for ESRD-RTT (Fig. 3a) [9, 16]. Based on the US average annual wage given the average age of working-age individuals with ADPKD in each disease stage (i.e., $51,166 for CKD stages 1–3, $51,856 for CKD stages 4–5, and $51,856 for ESRD-RRT) [36], total excess indirect costs associated with productivity loss from unemployment were estimated at $784 million.

Employed individuals with ADPKD experienced reduced productivity compared to the US employed population, with a productivity weight ranging from 91.3% for individuals with CKD stages 1–3 to 73.9% for individuals with ESRD-RRT (Fig. 3b) [9]. Based on the US average annual wage given the average age of working-age individuals with ADPKD in each disease stage, total excess indirect costs associated with productivity loss from reduced productivity at work were estimated at $390 million.

Premature mortality was also associated with a large productivity loss. Compared to the US population aged 35 years or older, annual all-cause mortality rates among individuals with ADPKD were 1.8 to 5.4 times higher depending on the age group, while the mortality rate was assumed to be equal for individual aged 15–34 years [15, 39, 40]. The productivity loss from premature mortality translated into total excess indirect costs estimated at $197 million.

Productivity loss associated with caregiving was estimated based on an average of 3.1, 27.0, and 46.7 h of caregiving annually for individuals with ADPKD with CKD stages 1–3, CKD stages 4–5, and ESRD-RRT, respectively [9], and a US average hourly wage of $24.34 [41]. Total excess indirect costs associated with productivity loss from caregiving were estimated at $53 million. Caregiving has also been associated with incremental direct healthcare costs [43]. Based on estimated increased direct healthcare costs of 8% for caregivers compared to those of non-caregivers (i.e., $434 per year), and a proportion of individuals with ADPKD with an unpaid caregiver estimated at 19% for CKD stages 1–5 and 57% for ESRD-RRT, total excess costs from caregivers’ direct healthcare costs were estimated at $17 million [29, 34, 43, 44].

### Sensitivity analyses

The main analysis presented the most conservative estimates of the economic burden associated with ADPKD (i.e., $7.3 billion; $51,970 per individual with ADPKD). In sensitivity analyses, the economic burden associated with ADPKD was estimated at up to $9.6 billion, that is, $68,091 per individual with ADPKD. By assuming that Medicare-insured individuals incur 14.4% higher direct healthcare costs than commercially insured individuals and that uninsured individuals with ADPKD incur 3 to 18 times higher direct healthcare costs than the uninsured US population (depending on disease stage), estimated total excess direct healthcare costs increased from $5.7 billion to $6.1 billion [10, 18, 46]. By varying the number of hours devoted to caregiving, indirect costs due to caregivers’ productivity loss increased from $53 million to $536 million as the number of hours devoted to caregiving for individuals with ADPKD increased to 3.1, 92.6, and 638.2 annually for individuals with CKD stages 1–3, CKD stages 4–5, and ESRD-RRT, respectively [9, 13, 47]. Finally, by varying the source used to estimate the prevalence of ADPKD per disease stage, the proportion of individuals with ADPKD with ESRD-RRT was increased from 22.7 to 32.2% [17], increasing the estimate of total excess direct healthcare costs to $7.2 billion and that of total excess indirect costs to $2.3 billion.

## Discussion

This study estimated the societal costs of ADPKD in the US in 2018 and found that ADPKD was associated with a significant burden. Indeed, incremental costs associated with ADPKD were estimated at $7.3 billion, equivalent to $51,970 per individual with ADPKD. In sensitivity analyses based on different assumptions, estimates ranged up to $9.6 billion, equivalent to $68,091 per individual with ADPKD. The largest contributor to excess costs of ADPKD was direct healthcare costs ($5.7 billion; 78.6% of total excess costs), over half of which were incurred by individuals with ESRD-RRT. Excess indirect costs were estimated at $1.4 billion (19.7% of total excess costs), driven primarily by costs of unemployment ($784 million; 10.7% of total excess costs) and reduced productivity at work ($390 million; 5.3% of total excess costs). Total excess direct non-healthcare costs were estimated at $125 million.

Prior studies reporting on the burden of ADPKD are scarce, with studies reporting both direct and indirect costs associated with ADPKD being based on non-US populations and studies based on the US population focusing on direct costs only. One study that examined both direct and indirect costs of ADPKD among a population from four Nordic countries (i.e., Denmark, Finland, Norway and Sweden) showed that the economic burden of ADPKD was substantial at all levels of disease. It also found that progression was associated with increased resource use and costs including direct healthcare costs and indirect costs related to productivity loss and informal care [9]. Depending on the country, direct healthcare costs comprised 25–43% of total costs associated with ADPKD among patients with CKD stages 1–3, 27–37% among those with CKD stages 4–5, and 60–90% among those with ESRD-RRT [9]. Indirect costs accounted for a substantial proportion of the burden of ADPKD (10–75% depending on disease stage and country), and were largely driven by productivity loss. However, the representativeness of these estimates in a US population may be limited given the important differences in the healthcare systems between the two populations, which may translate into different healthcare resource utilization and disease management.

In a US context, there is scant information on the burden of ADPKD from a societal perspective as prior studies estimating the costs of ADPKD in the US focused on direct healthcare costs only and did not include indirect costs [7, 10, 11]. Consistent with the current study, these studies found that total healthcare costs associated with ADPKD are high, particularly among patients with ESRD requiring dialysis [7, 10, 11]. More generally, in the CKD setting, studies in the US population have also shown that healthcare costs associated with CKD are substantial and increase steadily with each CKD stage progression, with ESRD costs being the highest [48, 49]. In a study published in 2017, excess annual healthcare costs associated with CKD in the US were estimated at $19,564 per individual with CKD, or $12.0 billion for the US population [49].

Studies focusing only on direct healthcare costs do not fully account for the societal burden associated with ADPKD, as indirect costs related to work productivity loss and caregiver support account for a significant proportion of overall costs in this population. Indeed, as this study found, indirect costs accounted for 19.7% of the burden associated with ADPKD in the US. Importantly, although rare diseases, by definition, have a low prevalence, they can nonetheless carry a significant overall economic burden, comparable to more common diseases, but one that translates to a greater cost per patient. For example, the total incremental cost of ADPKD estimated at $7.3 billion in the present study amounts to $51,970 per patient per year–nearly four times higher than that estimated for diabetes per patient ($13,504 per patient per year), another disease that can lead to CKD, with a higher prevalence compared to ADPKD [21]. In the context of rare diseases, examples of annual excess direct and indirect costs include $24,612 for juvenile idiopathic arthritis and $31,765 for scleroderma, which are also substantially lower than that for ADPKD found in the present study [50, 51].

Rare diseases in general are subject to high unmet needs in part due to limited knowledge of the disease and the challenges in conducting intensive research to develop therapeutic options for a rare patient population. Given the considerable burden per patient, any treatment strategy targeted at improving or delaying disease manifestations in ADPKD carries a high potential for reducing disease burden. Our findings showed that most of the burden associated with ADPKD was incurred by individuals with ESRD-RRT, suggesting that effective treatments and practices targeted at alleviating the manifestations of the disease and preventing decline of renal function have the potential to reduce healthcare costs in these patients, as well as indirect productivity loss and caregiver burden. In particular, as hospital readmissions have been shown to contribute to higher costs in patients with ESRD-RRT [48], efforts to reduce readmissions would lead to cost reductions. Moreover, effective management of increasing comorbidities with advancing disease stage, including heart failure and hyperkalemia, may offer further potential for cost reductions. Novel treatments for ADPKD are currently the subject of several clinical trials, and, in 2018, one treatment received regulatory approval in the US for the treatment of ADPKD in patients at risk of rapidly progressing disease [52–57]. These treatments have the potential help alleviate the burden of ADPKD for both individuals living with this disease, and their caregivers.

This study is subject to a number of limitations. Direct healthcare costs were calculated among individuals with a recorded diagnosis for ADPKD who had symptomatic disease (e.g., CKD), and thus may not fully reflect the costs incurred from a societal perspective as undiagnosed and/or asymptomatic individuals may have incurred costs which were not captured. Other costs may not have been captured in the current analysis. For example, there is scarce information on costs incurred by live kidney donors; therefore, these costs were not included. Similarly, costs associated with disability leaves may not have been fully captured under the productivity loss component as there was limited information on the extent to which individuals on disability leave were included in the studies used for the calculations. Another limitation is that there was scant information on direct excess healthcare costs incurred by individuals with ADPKD insured with Medicare or Medicaid, such that estimates of excess direct healthcare costs heavily relied on estimates among commercially insured individuals. While sensitivity analyses were conducted to account for potential differences between insurance types, further studies are warranted to confirm estimates for these populations. Moreover, there was little information on the number of hours devoted to caregiving by patient’s disease stage (especially in the US), and there were important variations in published estimates from different sources. While sensitivity analyses were conducted to account for various estimates, further studies are warranted to confirm the unpaid time devoted to caring for individuals with ADPKD in the US. Finally, in the absence of a single data source for costs related to ADPKD, several estimates from the literature and governmental publications were combined, including ex-US sources and/or sources pertaining to renal diseases (e.g., cystic kidney disease, CKD or ESRD in general) which may not be entirely representative of the US context. Similarly, while the costs incurred by patients with ADPKD should have ideally been compared to those of individuals with similar characteristics, identification of comparison groups of individuals without ADPKD was limited by the scope of the published literature and data from government and non-profit organizations. For example, the excess direct healthcare costs (representing ~ 75% of the total excess costs) were derived from a cohort of individuals with ADPKD and a sex- and age-matched cohort of individuals without ADPKD [10]. Yet, other differences between cohorts may not have been adjusted for, which may have confounded the estimates.

## Conclusion

ADPKD imposes substantial societal costs. Total annual excess costs associated with ADPKD were estimated at $7.3 billion, equivalent to $51,970 per individual with ADPKD in the US in 2018. While direct healthcare costs are the main drivers of the burden of ADPKD, indirect costs are substantial from a societal perspective and correspond to about 20% of the annual burden of ADPKD, with most of these costs incurred by individuals with ESRD-RRT. The findings of this study suggest that timely and effective treatment to alleviate the manifestations of the disease may significantly reduce the burden of ADPKD on individuals and society.

## Data Availability

Data sharing is not applicable to this article as no datasets were generated or analyzed during the current study. All information used for this study is available from published studies or reports, as cited throughout the Methods section.

## Abbreviations

ADPKD: Autosomal dominant polycystic kidney disease
CKD: Chronic kidney disease
CPI-U: Consumer Price Index for All Urban Consumers
ESRD: End-stage renal disease
RRT: Renal replacement therapy
USRDS: United States Renal Data System

## References

[1] Torres VE, Harris PC, Pirson Y (2007). Autosomal dominant polycystic kidney disease. Lancet.

[2] Helal I (2012). Prevalence of cardiovascular events in patients with autosomal dominant polycystic kidney disease. Am J Nephrol.

[3] Grantham JJ, Mulamalla S, Swenson-Fields KI (2011). Why kidneys fail in autosomal dominant polycystic kidney disease. Nat Rev Nephrol.

[4] Grantham JJ (2008). Clinical practice. Autosomal dominant polycystic kidney disease. N Engl J Med.

[5] National Institutes of Health (2010). Fact Sheet: Autosomal Dominant Polycystic Kidney Disease.

[6] Barnawi RA (2018). Is the light at the end of the tunnel nigh? A review of ADPKD focusing on the burden of disease and tolvaptan as a new treatment. Int J Nephrol Renovasc Dis.

[7] Blanchette CM (2015). Hospital-based inpatient resource utilization associated with autosomal dominant polycystic kidney disease in the US. J Med Econ.

[8] Brunelli SM (2015). End-stage renal disease in autosomal dominant polycystic kidney disease: a comparison of dialysis-related utilization and costs with other chronic kidney diseases. Clinicoecon Outcomes Res.

[9] Eriksson D (2017). Real-world costs of autosomal dominant polycystic kidney disease in the Nordics. BMC Health Serv Res.

[10] Knight T (2015). Medical resource utilization and costs associated with autosomal dominant polycystic kidney disease in the USA: a retrospective matched cohort analysis of private insurer data. Clinicoecon Outcomes Res.

[11] Lentine KL (2010). Renal function and healthcare costs in patients with polycystic kidney disease. Clin J Am Soc Nephrol.

[12] Stephens M (2013). High costs of Dialysis transportation in the United States: exploring approaches to a more cost-effective delivery system. JHEOR.

[13] Turchetti G (2017). The social cost of chronic kidney disease in Italy. Eur J Health Econ.

[14] Willey CJ (2018). The descriptive epidemiology of autosomal dominant polycystic kidney disease (ADPKD) in the United States, 2013–2015.

[15] US Census Bureau (2018). Population Estimate Program.

[16] United States Renal Data System (2017). USRDS annual data report: Epidemiology of kidney disease in the United States.

[17] Neumann HP (2013). Epidemiology of autosomal-dominant polycystic kidney disease: an in-depth clinical study for South-Western Germany. Nephrol Dial Transplant.

[18] Cloutier M (2018). The economic burden of bipolar I disorder in the United States in 2015. J Affect Disord.

[19] Greenberg PE, Stiglin LE, Finkelstein SN (1993). The economic burden of depression in 1990. J Clin Psychiatry.

[20] Stoudemire A (1986). The economic burden of depression. Gen Hosp Psychiatry.

[21] American Diabetes Association (2018). Economic Costs of Diabetes in the U.S. in 2017. Diabetes Care.

[22] American Diabetes, A (2013). Economic costs of diabetes in the U.S. in 2012. Diabetes Care.

[23] Goeree R (2005). The economic burden of schizophrenia in Canada in 2004. Curr Med Res Opin.

[24] Goeree R (1999). The economic burden of schizophrenia in Canada. Can J Psychiatr.

[25] Wu EQ (2005). The economic burden of schizophrenia in the United States in 2002. J Clin Psychiatry.

[26] Cloutier M (2016). The economic burden of schizophrenia in the United States in 2013. J Clin Psychiatry.

[27] US Department of Labor Bureau of Labor Statistics (2018). Consumer Price Index.

[28] Cubanski J, et al. How much is enough? Out-of-pocket spending among Medicare beneficiaries: a chartbook. 2014: The Henry J. Kaiser Family Foundation. Washington, DC. Publication # 8612.

[29] Center for Financing, Access, and Cost Trends, Agency for Healthcare Research and Quality (2015). Medical Expenditure Panel Survey.

[30] Small C (2017). Non-dialysis dependent chronic kidney disease is associated with high total and out-of-pocket healthcare expenditures. BMC Nephrol.

[31] PKD Foundation (2017). PKD Foundation Annual Report 2017.

[32] National Institute of Health Research Portfolio Online Reporting Tools (RePORT) (2018). Estimates of Funding for Various Research, Condition, and Disease Categories (RCDC).

[33] US Department of Health & Human Services (2016). OPTN/UNOS Registration Fee Increase First Time Since 2013.

[34] AARP Public policy institute and National Alliance for caregiving, Caregiving in the US, N.a.A.P.P. Institute, Editor. 2015.

[35] United Network for Organ Sharing (2015). UNOS Bylaws.

[36] US Bureau of Labor Statistics (2018). Labor Force Statistics.

[37] Organisation for Economic Co-operation and Development (2018). Employment Ratio by Sex and Age Group.

[38] Witt, E. and M. DiBonaventura, Work productivity loss and activity impairment across nineteen medical conditions in a representative sample of us adults. , P. Poster Presented at the ISPOR 20th Annual International Meeting, PA, USA, Editor. 2015.

[39] Hwang YH (2016). Refining genotype-phenotype correlation in autosomal dominant polycystic kidney disease. J Am Soc Nephrol.

[40] Munnell AH. The Average Retirement Age – An Update. Chestnut Hill: Center for Retirement Research at Boston College; 2015. Available from: http://crr.bc.edu/wp-content/uploads/2015/03/IB_15-4_508_rev.pdf; [27 March 2017].

[41] US Bureau of Labor Statistics (2017). United States Bureau of Labor Statistics.

[42] Greenberg PE (2015). The economic burden of adults with major depressive disorder in the United States (2005 and 2010). J Clin Psychiatry.

[43] Albert, S., R. Schulz, and A. Colombi*,* The MetLife study of working caregivers and employer healthcare costs, I.o.A. University of Pittsburg, National Alliance for Caregiving, MetLife Mature Market Institute., Editor. 2010.

[44] Suri RS (2011). Burden on caregivers as perceived by hemodialysis patients in the frequent hemodialysis network (FHN) trials. Nephrol Dial Transplant.

[45] Azzabi I, et al. Medicare claims analysis results on the medical costs associated with autosomal dominant polycystic kidney disease (ADPKD) in the US. Gaithersburg: Covance Market Access Services; 2011.

[46] Coughlin TA (2014). Uncompensated Care for Uninsured in 2013: A Detailed Examination. The Kaiser commission on Medicaid and the Unisured.

[47] Belasco AG, Sesso R (2002). Burden and quality of life of caregivers for hemodialysis patients. Am J Kidney Dis.

[48] Golestaneh L (2017). All-cause costs increase exponentially with increased chronic kidney disease stage. Am J Manag Care.

[49] Ozieh MN (2017). Trends in healthcare expenditure in United States adults with chronic kidney disease: 2002-2011. BMC Health Serv Res.

[50] Allaire SH (1992). The economic impacts of juvenile rheumatoid arthritis. J Rheumatol.

[51] Wilson L (1997). Cost-of-illness of scleroderma: the case for rare diseases. Semin Arthritis Rheum.

[52] Hogan MC (2010). Randomized clinical trial of long-acting somatostatin for autosomal dominant polycystic kidney and liver disease. J Am Soc Nephrol.

[53] Chebib FT, Torres VE (2018). Recent advances in the Management of Autosomal Dominant Polycystic Kidney Disease. Clin J Am Soc Nephrol.

[54] Caroli A (2013). Effect of longacting somatostatin analogue on kidney and cyst growth in autosomal dominant polycystic kidney disease (ALADIN): a randomised, placebo-controlled, multicentre trial. Lancet.

[55] Walz G (2010). Everolimus in patients with autosomal dominant polycystic kidney disease. N Engl J Med.

[56] US Food & Drug Administration (2018). Drug Approval Package: Jynarque (tolvaptan).

[57] Chebib FT (2018). A practical guide for treatment of rapidly progressive ADPKD with Tolvaptan. J Am Soc Nephrol.

